# A comparative study of diagnostic performance in shoulder pain: clinical tests versus ultrasonography and magnetic resonance imaging

**DOI:** 10.55730/1300-0144.6065

**Published:** 2025-08-31

**Authors:** Yasemin TOMBAK, Özgür Zeliha KARAAHMET, Ayşegül TOMBAK, Ömer ATA, Eda GÜRÇAY

**Affiliations:** 1 Department of Physical Medicine and Rehabilitation, University of Health Sciences, Ankara Etlik City Hospital, Ankara, Turkiye; 2 Department of Physical Medicine and Rehabilitation, Meram State Hospital, Konya, Turkiye; 3 Department of Radiology, University of Health Sciences, Ankara Etlik City Hospital, Ankara, Turkiye; 4 Department of Physical Medicine and Rehabilitation, University of Health Sciences, Gaziler Physical Medicine and Rehabilitation Training and Research Hospital, Ankara, Turkiye

**Keywords:** Magnetic resonance imaging, sensitivity, shoulder pain, shoulder-specific tests, specificity, ultrasonography

## Abstract

**Background/aim:**

Shoulder pain is common and often evaluated with physical examination tests, yet their diagnostic accuracy remains controversial. The aim of this cross-sectional study was to determine the sensitivity, specificity, positive predictive value (PPV) and negative predictive value (NPV) rates of clinical test results in patients with shoulder pain, based on ultrasonography (US) and magnetic resonance imaging (MRI) findings as reference diagnostic methods.

**Materials and methods:**

The study included 78 patients who had complaints of shoulder pain. All patients underwent physical examinations and specific clinical tests (such as Neer and Jobe tests) to reveal shoulder pathologies, and US and MRI evaluations were performed.

**Results:**

The sensitivity of clinical examination tests for subacromial impingement syndrome, biceps tendinitis and rupture, and infraspinatus tendinitis was higher than US. The specificity of tests for biceps rupture, rotator cuff rupture, infraspinatus and subscapularis tendinitis was higher compared with US. Subacromial impingement tests also showed higher sensitivity compared to MRI. The specificity of tests for acromioclavicular arthritis, biceps rupture, rotator cuff rupture, infraspinatus tendinitis was higher than MRI. Comparison of diagnostic performance with US was significant for subacromial impingement syndrome, acromioclavicular arthritis, biceps tendinitis and rupture, rotator cuff rupture and subscapularis tendinitis (p values; 0.003, 0.003, 0.000, 0.026, 0.000, 0.000, respectively). In contrast, comparison with MRI was significant only for subacromial impingement syndrome (p = 0.026).

**Conclusion:**

Clinical tests have an important place in practice when examining shoulder lesions with their high specificity and sensitivity. Although US reveals many shoulder lesions that cannot be detected by clinical tests or support our examination, MRI maintains its importance in shoulder imaging.

## 1. Introduction

Twenty percent of people worldwide suffer from shoulder pain [1]. Approximately two-thirds of individuals with non-specific shoulder pain have a rotator cuff lesion; however, tendinosis, adhesive capsulitis, osteoarthritis, labral tears, and transferred pain from the neck are all possible causes [2]. Physical examination tests are primarily preferred in diagnosis. These tests aim to simulate particular symptoms and indicators. The literature has described a wide variety of tests, so it can be challenging to decide which test to use [3]. The same test was conducted using various positivity criteria. For example, ‘weakness’ [4] and/or ‘pain’ [5] were used as positivity criteria for the supraspinatus test. Additionally, when the same test is called by different names (e.g., Supraspinatus test = Empty can test = Jobe test), confusion results [4–6]. The Yergason test [7], which measures biceps pathology, is also utilized to assess glenoid labral pathology [8].

The results of specific shoulder tests compared with imaging methods can give us an idea of which preliminary diagnoses will require imaging. There are studies in literature comparing US, MRI and clinical findings, but the issue of which clinical test has more diagnostic sensitivity is still controversial [9–11]. To date, no studies in the literature have been identified that combined specific shoulder tests, US, and MRI in the same patient. Therefore, we aimed to determine the sensitivity, specificity, positive predictive value (PPV), and negative predictive value (NPV) of clinical test results based on US and MRI in patients with shoulder pain.

## 2. Materials and methods

### 2.1. Study design and patient recruitment

The study was carried out in compliance with the Declaration of Helsinki’s principles and with authorization from the local ethics committee (Protocol No: 2023-695, dated 22.11.23). Every patient who took part in the study provided written consent.

This cross-sectional study included 78 consecutive patients aged 25–80 years who applied to the Physical Medicine and Rehabilitation (PMR) outpatient clinic with complaints of shoulder pain between January 2024 and April 2024.

The physician performing the clinical tests was blind to the US and MRI images. Physicians performing US and evaluating MRI images were blind to physical examination findings. US was performed within 3 days following the physical examination.

### 2.2. Exclusion and inclusion criteria

Patients over the age of 18 who had an MRI with shoulder pain in the last 2 weeks and agreed to participate in the study were included. Patients with a history of trauma or surgery, inflammatory rheumatic disease, another musculoskeletal problem in the upper extremity, and limited passive joint movements were excluded from the study.

**Figure 1:**
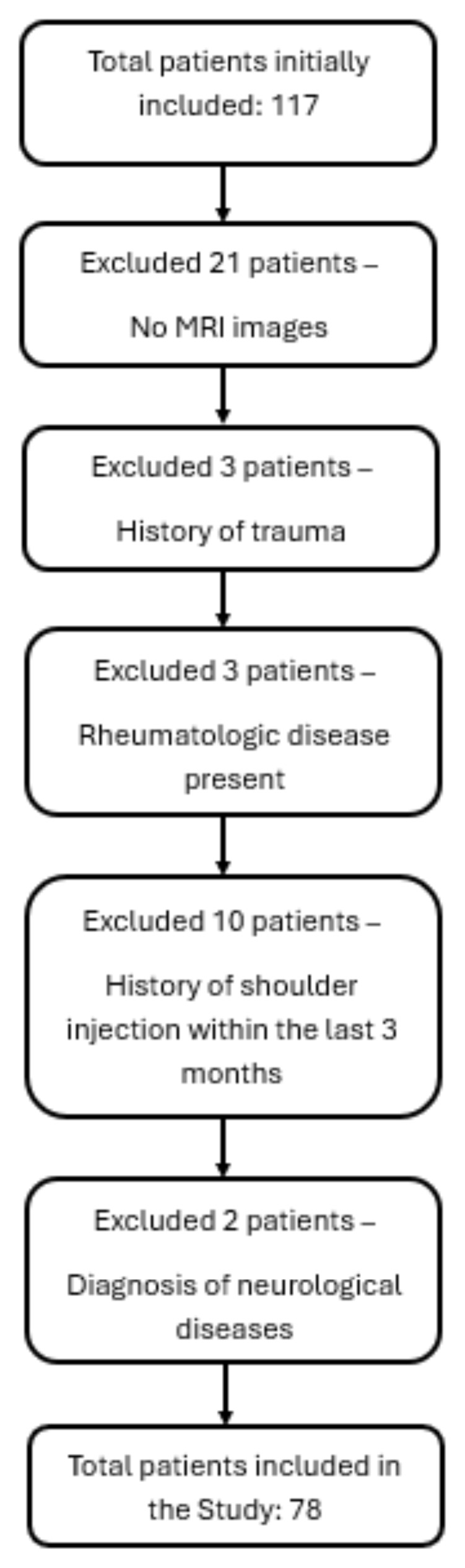
Flow diagram of patient enrollment and exclusion.

The flow diagram illustrating patient enrollment and exclusion criteria is presented in Figure 1.

### 2.3. Clinical evaluation

The patients’ age, gender, education level, comorbidity, dominant side, symptomatic side, and symptom duration were recorded.

Specific shoulder tests and associated shoulder pathologies are shown in Table 1.

### 2.4. Ultrasonographic evaluation

Sonographic examinations were carried out in all patients with a single physician who had at least 5 years of US experience in musculoskeletal sonography blinded to the clinical test results of the patients, using a 7–15 MHz linear-array transducer (LOGIQ 9, GE HealthCare, Wauwatosa, WI, USA).

There have been many written accounts of the US examination method for the shoulder in the literature [30–32]. Scanning was performed while the subjects were in a sitting position, care was taken to ensure that the examined arm was not in abduction position, and the imaging included the humeral head’s circular structure.

The patient was positioned so that the elbow was flexed to 90° and the forearm was half pronated on the lap when the biceps tendon was examined. The long head of the biceps tendon is imaged as an oval-shaped echogenic structure on the anterior aspect of the shoulder. With a small external rotation of the GH joint, the hyperechoic subscapularis tendon was located anteromedial to the biceps tendon (Figure 2a). The patient’s shoulder was placed in hyperextension and full internal rotation while the dorsum of the hand was placed in the small of the back to examine the supraspinatus tendon. In a longitudinal view, supraspinatus tendon appears as a convex, tapered, hyperechoic fibrillar layer that inserts at the greater tuberosity (Figure 2b). The bursa subacromial-subdeltoid was detected on imaging as a hypoechoic line situated between the supraspinatus tendon and the deltoid muscle (Figure 2c). Patient’s hand was placed on the contralateral shoulder for examination of the Glenohumeral (GH) joint and infraspinatus tendon.

### 2.5. Magnetic resonance imaging evaluation

Axial Proton Density (PD), coronal PD-T2 fat suppressed (FS), coronal T1 Fast Spin Echo (FSE), and sagittal PD FS sequences were used in all patients. Rotator cuff tendons were examined for tendinitis, partial and full-thickness rupture. Increased signal intensity in PD-weighted sections was evaluated as tendinitis, increased fluid intensity in articular, bursal or intratendinous areas in T2 was evaluated as partial rupture, focal or diffuse loss of integrity and retraction in the tendon was evaluated as full-thickness rupture. Subacromial-subdeltoid bursa, glenohumeral joint, biceps tendon, acromioclavicular joint, humeral head changes were examined routinely.

**Figure 3:**
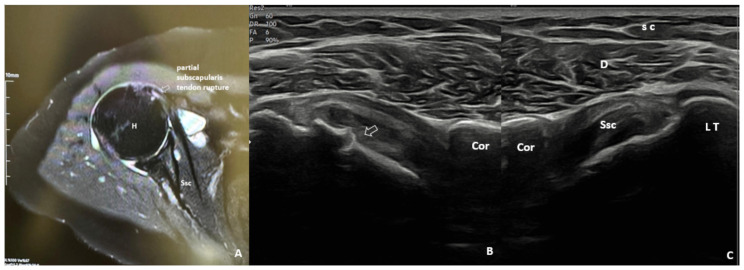
Axial MRI image demonstrating a partial rupture of the subscapularis tendon **(A)** Axial US image over the anteromedial shoulder reveals a partial subscapularis tendon rupture possibly due to the underlying cortical irregularities (arrow) of the lesser tuberosity **(B)** US image of normal contralateral side **(C)**. Cor: Coracoid process; D: Deltoid; H: Humerus; LT: Lesser tuberosity; sc: Subcutaneous tissue; Ssc: Subscapularis tendon.

**Figure 4:**
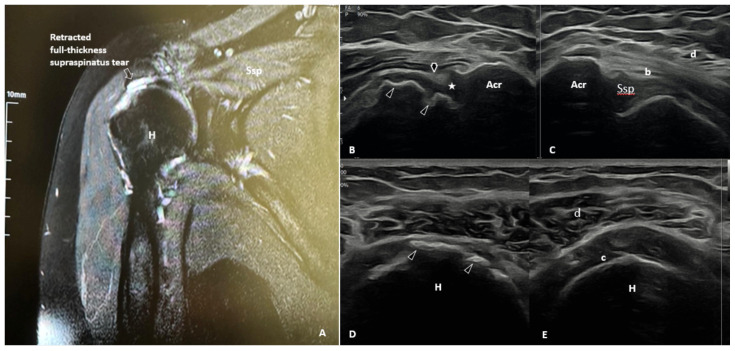
Coronal MRI image showing a full-thickness retracted tear (arrow) of the supraspinatus tendon **(A)** Longitudinal **(B)** and axial **(D)** US images illustrate a full-thickness tear of the supraspinatus tendon (arrow) and cortical irregularities (arrowheads) of the humerus. Lateral coronal **(B)** US image shows the absence of “bird’s beak” appearance (star) of the supraspinatus tendon **(B)**. Corresponding contralateral views show a normal appearance of the supraspinatus tendon **(C, E)**. Acr: Acromion; b: Subdeltoid bursa; c: Cartilage; d: Deltoid muscle; H: Humerus; Ssp: Supraspinatus tendon

Shoulder US and MRI images of the same patient are shown in Figure 3 and Figure 4.

### 2.6. Statistical analysis

All the statistical analyses were performed using Statistical Package for the Social Sciences (SPSS) for Windows (Version 22.0, IBM Corp., Chicago, IL, U.S.A.). With regard to categorical variables, data are displayed as frequency (%). When calculating a p value for categorical variables, the chi-squared test was used. Statistics were deemed significant if the p-value was less than 0.05.

### 2.7. Sample size calculation

The sample size calculations were based on data from the earlier study by Naredo et al. [31]. The number of participants was calculated using an incidence of subacromial impingement syndrome population (44%) and study group (20%) respectively, with 95% power and 1% significance. We eventually determined that at least 65 people would be allocated.

## 3. Results

Demographic and clinical data of the patients are shown in Table 2. The average age was found to be 53.5 years and 74.4% were female.

Table 3 demonstrates the distribution of the patients’ clinical, sonographic, and MRI-detected shoulder lesions. Tables 4 and 5 show the diagnostic performance levels of clinical test results in distinguishing patients with and without shoulder lesions detected by US and MRI, respectively.

When clinical examination results of subacromial impingement syndrome, biceps tendinitis and rupture, and infraspinatus tendinitis were compared with US, their sensitivity was found to be high.

When clinical examination results for biceps rupture, rotator cuff rupture, infraspinatus and subscapularis tendinitis were compared with US, their specificity was found to be high.

Subacromial impingement syndrome was found to have higher sensitivity when clinical examination results were compared with MRI.

When clinical examination results for AC arthritis, biceps rupture, rotator cuff rupture, infraspinatus tendinitis were compared with MRI, their specificity was found to be high.

## 4. Discussion

In this study diagnostic performance levels of clinical tests were investigated with the sensitivity, specificity, PPV and NPV parameters based on US and MRI results in shoulder pain. The most clinically detected cases were subacromial impingement syndrome (51.3%) and supraspinatus tendinitis (51.3%). Supraspinatus tendinitis was mostly detected in MRI and US. The most common occurrence of supraspinatus tendinitis was consistent with the literature [33].

One of our remarkable results was that, contrary to the literature [34], subscapularis tendinitis was detected more frequently as examination, US and MRI findings than infraspinatus tendinitis.

According to our results, we found that the sensitivity of clinical tests compared to US in subacromial impingement syndrome was 91.7% and the negative predictive value was 97.4%. Again, the sensitivity and negative predictive value of the examination in subacromial impingement syndromes were as high when compared with MRI as they were when compared with US. In addition, the specificity of clinical tests compared to US in subacromial impingement syndrome was found to be 56.1% and PPV was 27.5%. The reason why we found the specificity to be low while the sensitivity was high may be that we applied the tests in combination. Calis et al. in their study examined combinations of clinical tests and found that as the number of tests found positive together increased, sensitivity rates increased, and specificity rates decreased [35].

When clinical tests were compared with US in AC arthritis, sensitivity was found to be 25%, specificity was 88%, positive predictive value was 100%, and negative predictive value was 88%. The fact that we performed a single test instead of a combined test in AC arthritis may have caused the specificity to be high. This situation in AC arthritis was also present in the clinical examination and MRI comparison. While physical examination tests were not sensitive compared to MRI in AC arthritis, a specificity of 95% was detected.

In a study comparing US and MRI findings, the sensitivity and specificity of US in full-thickness tears of the supraspinatus tendon were found to be 95%, in cases of partial tears the sensitivity was 91% and specificity was 98%, in cases of supraspinatus tendinosis the sensitivity and specificity were found to be 90% and specificity 93% [36]. Our sensitivity and specificity results of physical examination compared to MRI in supraspinatus partial tear and tendinitis were lower than the results of this study. The reason we over-detect supraspinatus tendinitis during examination may be that it is also positive in other shoulder pathologies. During clinical tests, pain occurs by stimulating nociceptors by compressing or stretching the subacromial bursa. Weakness in the arm occurs due to pain inhibition, and the clinician may misinterpret these findings. This may explain why the tests show false positivity [11].

In addition, although we did not detect any full-thickness tears of the supraspinatus tendon during the examination, it was detected in the MRI. We could not detect any subscapularis or infraspinatus tears on examination. With these results, we do not know whether physical examination is insufficient to detect tendon ruptures or whether the results vary from person to person. We can emphasize the importance of MRI in detecting full-thickness tendon tears. Clinical tests may suffice for the initial evaluation, particularly for subacromial impingement, but MRI should be prioritized when tendon ruptures are suspected. In a study where shoulder examination and US evaluations were performed in patients with spinal cord injury who had shoulder pain, Neer’s impingement test was the most common examination finding, while O’Brien’s test was the least common examination finding. Sensitivity-specificity evaluation was not performed in this study [37]. In our case, subacromial impingement syndrome was easily detected dynamically using US. However, superior labrum anterior and posterior (SLAP) lesions were detected less frequently. Subacromial impingement syndrome is a dynamic pathology. For this reason, MRI may not be preferred in detecting subacromial impingement syndrome. We can easily detect subacromial impingement with dynamic impingement findings on US [38].

In Günay’s study, the Speed test was found to be 100% sensitive in biceps tendinitis, and the specificity was found to be 13.6%. Our study differs in that we evaluated biceps tendinitis using both the Speed and Yergason tests [10]. While the sensitivity of clinical tests in biceps pathologies is higher in the literature, we found the specificity to be high [11,35,39].

Leroux et al. found the sensitivity of the Patte test in infraspinatus lesions to be 92% and the specificity to be 30% [40]. While the diagnostic performance level of the Patte test in infraspinatus lesions was found to be statistically significant, the resisted rotation test was not found to have sufficient diagnostic performance [11]. Our results in infraspinatus tendinitis did not have significant diagnostic performance.

In one study, the lift-off test was found to be 89.47% sensitive in subscapularis partial tear [10]. We did not evaluate partial tears of the subscapularis because they could not be detected on clinical examination; however, we found that clinical tests had lower sensitivity than MRI in diagnosing subscapularis tendinitis. Clinically, we determined that the tests we performed for subscapularis tendinitis had high sensitivity, specificity, PPV and NPV rates, and significant diagnostic performance when compared to US. The higher number of subscapularis tendinitis cases in our study may have been effective in reaching this conclusion.

The results of Naredo et al. show that clinical diagnosis of periarticular conditions in the painful shoulder is not very accurate compared to US diagnosis [31]. They said that this was an expected result. They explained this situation as the impingement syndrome in most patients with chronic shoulder pain and the presence of several periarticular lesions, usually involving different tendons and the subacromial-subdeltoid bursa. In clinical tests, significant diagnostic performance was detected in subacromial impingement syndrome, AC arthritis, biceps tendinitis, biceps rupture, rotator cuff rupture and subscapularis tendinitis according to US.

### 4.1. Limitations and advantages of the study

There may be false positives in the examination, or there may be shoulder lesions that we cannot detect. In our study, supraspinatus full-thickness tears, biceps subluxation, SLAP, infraspinatus and subscapularis tears were detected on imaging, although they were not detected on examination.

We have not found many studies evaluating the sensitivity and specificity of clinical examination tests compared to US. Generally, studies were conducted with MRI evaluation. We compared clinical tests with both US and MRI. To the best of our knowledge, this is the first study to investigate the diagnostic performance of clinical tests by comparing two different imaging methods.

The single-center nature of our study, the relatively small sample size, and the operator dependency of US can be considered as limitations.

## 5. Conclusion

Patients with shoulder pain may have more than one pathological lesion. Clinical tests have an important place in practice when examining shoulder lesions with their high specificity and sensitivity. Although US reveals many shoulder lesions that cannot be detected by clinical tests or support our examination, MRI maintains its importance in shoulder imaging.

## Figures and Tables

**Figure 2a:**
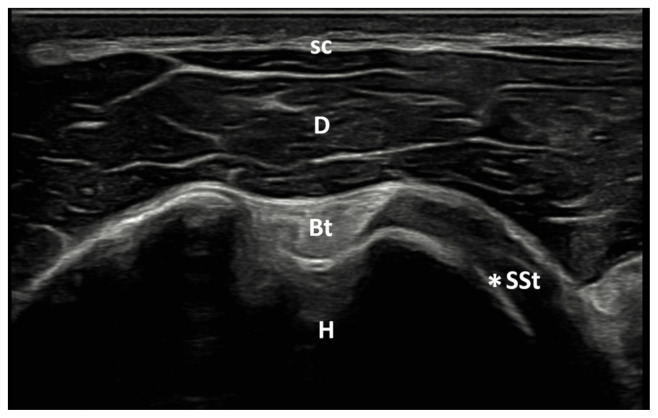
Anterior axial US image of the biceps tendon. Bt: Biceps tendon; D: Deltoid muscle; H: Humerus; sc: Subcutaneous tissue; *SSt: Subscapularis tendon

**Figure 2b:**
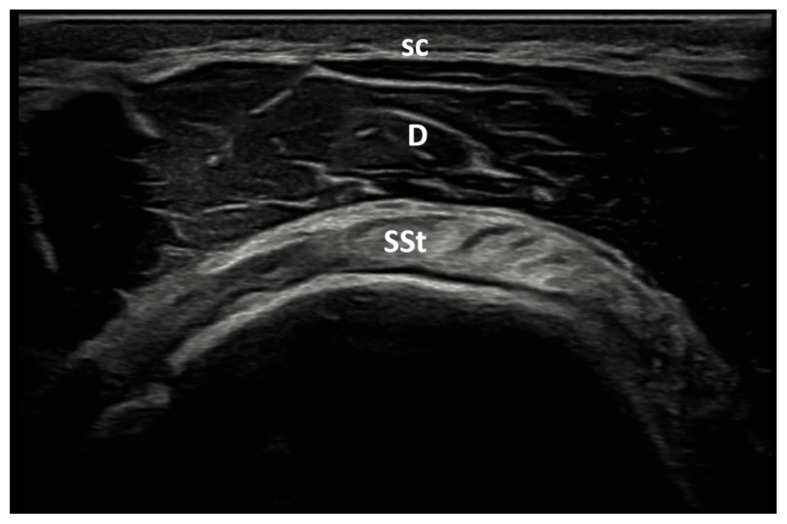
Axial US image of the rotator cuff in crass position. D: Deltoid muscle; sc: Subcutaneous tissue; SSt: Supraspinatus tendon

**Figure 2c:**
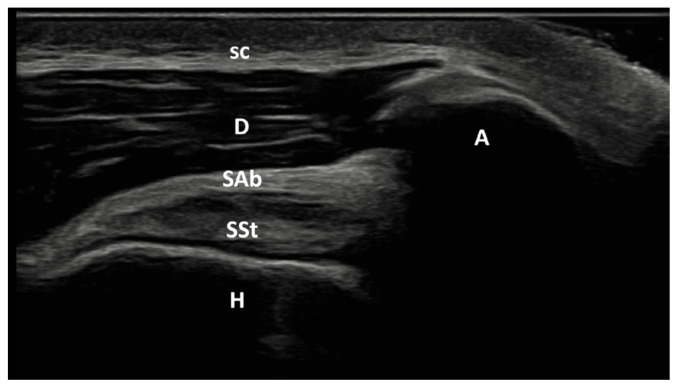
Lateral longitudinal US image of supraspinatus tendon. A: Acromion; D: deltoid muscle; H: Humerus; sc: Subcutaneous tissue; Sab: subacromial bursa; SSt: Supraspinatus tendon

**Table 1:** Specific shoulder tests and associated shoulder pathologies.

Conducted tests	Shoulder pathology
Neer test [12,13] Hawkins test [14] Yocum test [15] Painful arch test [16]	Subacromial impingement syndrome
Jobe test [6] Full can test [17] Drop arm test [18,19]	Supraspinatus tendon pathologies
Patte test [19] Resisted external rotation test [14] External rotation failure sign [20,21]	Infraspinatus tendon pathologies
Gerber Lift off test [22–24] Internal rotation failure sign [22–24] Abdominal compression test [22–25]	Subscapularis tendon pathologies
Speed test [26] Yergason test [27] Popeye sign [28]	Biceps tendon pathologies
Cross Body Adduction Stress Test [29]	Acromioclavicular joint pathologies.

**Table 2:** Demographic and clinical variables of the patients.

		n = 78, n (%), mean (SD)
Age, years		53.5 (10.2)
Sex	Female	58 (74.4)
	Male	20 (25.6)
Education level	Illiterate	7 (9)
	Primary school	38 (48.7)
	Middle school	7 (9)
	High school	14 (17.9)
	College	3 (3.8)
	University	9 (11.5)
Comorbidity	DM	16 (20.5)
	HT	17 (21.8)
	HL	5 (6.4)
	Thyroid function disorder	7 (9)
	Renal disease	3 (3.8)
	Heart valve disease	2 (2.6)
	Arrhythmia	1 (1.3)
	COPD	1 (1.3)
Dominant side	Right	76 (97.4)
	Left	2 (2.6)
Symptomatic side	Right	50 (64.1)
	Left	28 (35.9)
Symptom duration	3–6 months	29 (37.2)
	6–12 months	12 (15.4)
	>12 months	37 (47.4)

DM: Diabetes mellitus; HT: Hypertension, HL: Hyperlipidemia; COPD: Chronic Obstructive pulmonary disease

**Table 3:** Distribution of shoulder lesions detected clinically, ultrasonographically and by MRI.

	Clinically positive n (%)	Sonographically positive n (%)	Positive on MRI n (%)
Subacromial impingement	40 (51.3)	12 (15.4)	6 (7.7)
AC arthritis	3 (3.8)	12 (15.4)	11 (14.7)
Supraspinatus partial tear	13 (16.7)	17 (21.8)	28 (35.9)
Supraspinatus full thickness tear	0 (0)	1 (1.7)	6 (7.7)
Supraspinatus tendinitis	40 (51.3)	24 (30.8)	30 (38.5)
Biceps tendinitis	29 (37.2)	16 (20.5)	11 (11.7)
Biceps rupture	2 (2.6)	1 (1.3)	2 (2.6)
Biceps subluxation	0 (0)	1 (1.3)	0 (0)
Rotator cuff tear	3 (3.8)	4 (5.1)	1 (1.7)
SLAP	0 (0)	1 (1.3)	3 (3.8)
Infraspinatus tendinitis	4 (5.1)	1 (1.3)	7 (9)
Infraspinatus tear	0 (0)	0 (0)	2 (2.6)
Subscapularis tendinitis	17 (21.8)	19 (24.4)	9 (11.5)
Subscapularis tear	0 (0)	0 (0)	2 (2.6)

MRI: Magnetic resonance imaging, AC: Acromioclavicular; SLAP: Superior labrum anterior and posterior

**Table 4:** Diagnostic performance levels of clinical test results in patients with and without shoulder lesions detected by US.

Clinical examination results	Sensitivity	Specificity	PPV	NPV	p
Subacromial impingement	91.7	56.1	27.5	97.4	**0.003**
AC arthritis	25	88	100	88	**0.003**
Supraspinatus partial tear	23.5	85.2	30.8	80	0.464
Supraspinatus tendinitis	66.7	55.6	40	78.9	0.088
Biceps tendinitis	93.8	77.4	51.7	98	**0.000**
Biceps rupture	100	98.7	50	100	**0.026**
Rotator cuff rupture	75	100	100	98.7	**0.000**
Infraspinatus tendinitis	100	96.1	25	100	0.051
Subscapularis tendinitis	84.2	98.3	94.1	95.1	**0.000**

PPV: Positive predictive value; NPV: Negative predictive value, AC: Acromioclavicular

**Table 5:** Diagnostic performance levels of clinical test results in patients with and without shoulder lesions detected by MRI.

Clinical examination results	Sensitivity	Specificity	PPV	NPV	p
Subacromial impingement	100	52.8	15	100	**0.026**
AC arthritis	0	95.5	0	85.3	0.6
Supraspinatus partial tear	25	88	53.8	67.7	0.2
Supraspinatus tendinitis	56.7	52.1	42.5	65.8	0.4
Biceps tendinitis	27.3	61.2	10.3	83.7	0.5
Biceps rupture	50	98.7	50	98.7	0.051
Rotator cuff rupture	0	96.1	0	98.7	0.9
Infraspinatus tendinitis	14.3	95.8	25	91.9	0.3
Subscapularis tendinitis	44.4	81.2	23.5	91.8	0.09

PPV: Positive predictive value; NPV: Negative predictive value, AC: Acromioclavicular
